# Decreased level of serum NT-proCNP associates with disease severity in COVID-19

**DOI:** 10.1186/s12931-023-02469-4

**Published:** 2023-06-29

**Authors:** Istvan Bojti, Anne-Sophie Przewosnik, Hendrik Luxenburger, Maike Hofmann, Christoph Neumann-Haefelin, Jennifer S. Esser, Patrick M. Siegel, Alexander Maier, Sarolta Bojtine Kovacs, Laszlo Kardos, Zoltan Csanádi, Marina Rieder, Daniel Duerschmied, Achim Lother, Christoph Bode, Gabor Tamas Szabó, Daniel Czuriga

**Affiliations:** 1 Department of Cardiology and Angiology, University Heart Center, Medical Center-University of Freiburg, Faculty of Medicine, University of Freiburg, Freiburg, Germany; 2 Department of Medicine II (Gastroenterology, Hepatology, Endocrinology and Infectious Diseases), Medical Center-University of Freiburg, Faculty of Medicine, University of Freiburg, Freiburg, Germany; 3 IMM-PACT, Faculty of Medicine, University of Freiburg, Freiburg, Germany; 4 Section of Molecular Hematology, Department of Medicine I, Hematology, Oncology and Stem Cell Transplantation, Medical Center-University of Freiburg, Faculty of Medicine, University of Freiburg, Freiburg, Germany; 5 Clinical Department of Infectious Diseases, Clinical Center, University of Debrecen, Debrecen, Hungary; 6 Division of Cardiology, Department of Cardiology, Faculty of Medicine, University of Debrecen, Debrecen, Hungary; 7 Department of Cardiology, Angiology, Haemostaseology and Medical Intensive Care, University Medical Centre Mannheim, Medical Faculty Mannheim, Heidelberg University, Heidelberg, Germany; 8 European Center for AngioScience (ECAS) and German Center for Cardiovascular Research (DZHK) Partner Site Heidelberg/Mannheim, Mannheim, Germany; 9 Institute of Experimental and Clinical Pharmacology and Toxicology, Faculty of Medicine, University of Freiburg, Freiburg, Germany; 10 Interdisciplinary Medical Intensive Care, Medical Center-University of Freiburg, Faculty of Medicine, University of Freiburg, Freiburg, Germany

**Keywords:** COVID-19, SARS-CoV-2, NT-proCNP, Furin

## Abstract

**Background:**

C-type natriuretic peptide (CNP) is an endothelium-derived paracrine molecule with an important role in vascular homeostasis. In septic patients, the serum level of the amino-terminal propeptide of CNP (NT-proCNP) shows a strong positive correlation with inflammatory biomarkers and, if elevated, correlates with disease severity and indicates a poor outcome. It is not yet known whether NT-proCNP also correlates with the clinical outcome of patients suffering from severe acute respiratory syndrome coronavirus 2 (SARS-CoV-2) infection. In the current study, we aimed to determine possible changes in the NT-proCNP levels of patients with coronavirus disease 2019 (COVID-19), with special regard to disease severity and outcome.

**Methods:**

In this retrospective analysis, we determined the serum level of NT-proCNP in hospitalized patients with symptoms of upper respiratory tract infection, using their blood samples taken on admission, stored in a biobank. The NT-proCNP levels of 32 SARS-CoV-2 positive and 35 SARS-CoV-2 negative patients were measured to investigate possible correlation with disease outcome. SARS-CoV-2 positive patients were then divided into two groups based on their need for intensive care unit treatment (severe and mild COVID-19).

**Results:**

The NT-proCNP was significantly different in the study groups (e.g. severe and mild COVID-19 and non-COVID-19 patients), but showed inverse changes compared to previous observations in septic patients: lowest levels were detected in critically ill COVID-19 patients, while highest levels in the non-COVID-19 group. A low level of NT-proCNP on admission was significantly associated with severe disease outcome.

**Conclusions:**

Low-level NT-proCNP on hospital admission is associated with a severe COVID-19 disease course. The pathomechanism underlying this observation remains to be elucidated, while future studies in larger patient cohorts are necessary to confirm these observations and reveal therapeutic importance.

*Trial registration* DRKS00026655 Registered 26. November 2021

**Supplementary Information:**

The online version contains supplementary material available at 10.1186/s12931-023-02469-4.

## Background

The natriuretic peptides are potent modulators of vascular homeostasis [1]. Besides their central role in the regulation of fluid/electrolyte balance, vascular tone and cardiac remodelling [2], the amino terminals of both atrial natriuretic peptide (ANP; NT-proANP) and brain natriuretic peptide (BNP; NT-proBNP) are elevated in septic patients, most probably due to depressed myocardial function and ventricular dilatation, which represent septic heart failure (HF) signalling. Moreover, NT-proBNP can be used as a biomarker to predict survival in patients with severe sepsis [3]. In addition, the mid-region fragment of the proANP (MR-proANP) also emerged as a valuable prognostic marker of outcome in a broad range of clinical conditions, such as sepsis, heart failure or pneumonia [4–6]. As newly discovered, the elevation of both NT-proBNP and MR-proANP predicts a worse outcome in COVID-19 [7, 8].

C-type natriuretic peptide (CNP) has been proven as a predictor of poor clinical outcome in critically ill patients [9–11], since CNP upregulation is induced by proinflammatory cytokines including interleukin (IL) 1α, IL-1β, TNFα, by physical factors, such as shear stress or hypoxemia, or directly by bacterial endotoxins [12]. CNP however, has an important anti-inflammatory effect and also inhibits platelet reactivity [13].

As natriuretic peptides are synthetized in the form of prepropeptides, they need further proteolytic processing to be converted into their biologically active form [14]. Regarding CNP, furin has a crucial role in the cleavage of proCNP into NT-proCNP and the active CNP peptide. Without the intracellular proteolytic activation by furin, CNP cannot be secreted; therefore, it cannot fulfil its role in maintaining homeostasis [15]. Due to the short serum half-life of the CNP peptide, the measurement of the NT sequence with a longer half-life (also secreted following furin proteolysis) has found its way to clinical application [16].

Furin is a ubiquitous endoprotease with numerous substrates such as cytokines, growth factors, hormones, adhesion molecules, coagulation factors, membrane channels and albumin. In animal models, the lack of furin leads to an early death of the embryo due to severe developmental abnormalities [17].

Certain pathogens including human immunodeficiency virus (HIV), Ebola virus, measles virus, respiratory syncytial virus (RSV) also utilise a furin-mediated proteolysis, in particular for cellular entry [18–20]. Similarly, severe acute respiratory syndrome coronavirus 2 (SARS-CoV‐2) extends the list of viruses using furin cleavage for cellular entry [18]. However, to our current knowledge, there are no clinical data available exploring the possible correlation between the serum levels of furin, CNP homeostasis and disease outcome in SARS-CoV-2 infection. Given the mutual utilisation of furin by both proCNP and SARS-CoV-2, there may be a competition for furin-mediated cleavage during SARS-CoV-2 infection. Thus, the investigation of serum NT-proCNP levels in COVID-19 is not only scientifically intriguing, but also paramount from a clinical point of view in the era of ongoing COVID-19 pandemic. Here, we aimed at characterizing the changes in NT-proCNP levels in COVID-19 patients, with special regard to disease severity and outcome.

## Methods

### Study subjects

**Fig. 1:**
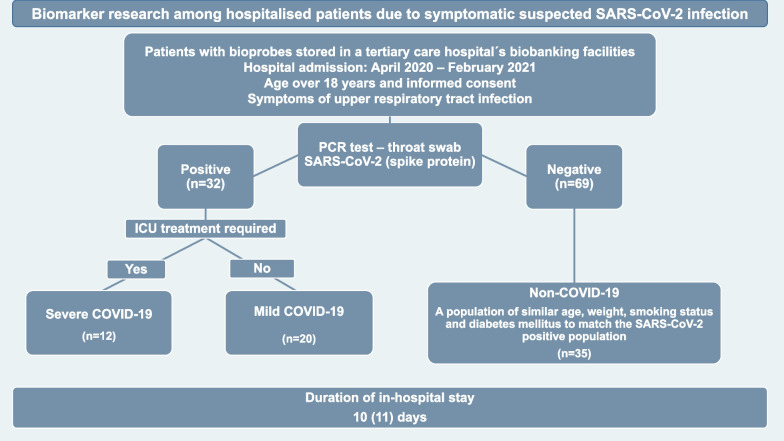
Study design and patient flow

In our retrospective study, we included patients from the biobank facility of the tertiary care hospital of the University of Freiburg, who were admitted with upper respiratory tract symptoms, raising the suspicion of SARS-CoV-2 infection, to the Department of Emergency Medicine and Interdisciplinary Intensive Care Unit between April 2020 and February 2021. The results of PCR tests for SARS-CoV-2 (spike protein) taken on admission were used to determine the presence of infection, and divide the patients accordingly. The thirty-two patients with proven SARS-CoV-2 infection were further divided based on their need for intensive care unit (ICU) treatment: 20 patients in need of hospitalization but no ICU treatment were allocated into the mild COVID-19, while 12 patients in need of ICU treatment into the severe COVID-19 subgroups. In the patient cohort tested negative for SARS-COV-2, a population of similar age, sex, smoking status, diabetes and body weight was selected (non-COVID-19 group) to match patients with proven SARS-CoV-2 infection. The flow chart of patients’ inclusion criteria is depicted in Fig. 1.

All patients have written informed consent for sample and data collection, plasma storage and analysis. None of the patients were vaccinated against SARS-CoV-2. Patients’ clinical data and laboratory data except NT-proCNP and furin results were obtained from the medical database. Deep-frozen blood samples were obtained from the biobank facility, which were analysed with commercially available ELISA kits for NT-proCNP (Enzo Life Sciences, Lausen, Switzerland) and furin (Hölzel Diagnostika, Köln, Germany), according to the manufacturer’s instructions. Primary endpoint for severe outcome was defined as a composite of death and/or need for mechanical ventilatory support and/or extracorporeal membrane oxygenation (ECMO). The single components of the primary endpoint (death, need for mechanical ventilatory support and/or ECMO) were secondary outcome measures.

### Statistical analysis

Continuous variables were described using mean and standard deviation (SD) for symmetric, and median and interquartile range (IQR) for asymmetric distributions. Unadjusted between-groups comparisons were based on two-sample t tests, if distributional assumptions were satisfied; otherwise, Wilcoxon’s rank-sum test was used. Categorical variables were described using absolute and relative frequencies; unadjusted between-groups comparisons were made using Fisher’s exact test.

For regression analysis, continuous variables were natural log-transformed if this improved distributional symmetry. Simple or multiple logistic regression was used to estimate the effects of predictors (clinical and laboratory parameters) on the composite primary endpoint and on secondary outcomes. Linearity of association with continuous predictors was examined and a quadratic term was introduced if this substantially improved model fit. In multiple logistic regression, we adjusted the estimates for age and sex on an a priori basis. We also assessed all other potential confounders to see if adjustment for them was necessary. No variable proved useful or possible to adjust for, due to one or more of the following reasons: lack of association with outcome, explanatory variable, or both; high proportion of missing values; addition to model causes perfectly determined outcomes; no change to other effect estimates or precision; change limited to decrease in precision (confounder being a quasi-proxy of the explanatory variable). Effect estimates were expressed as log odds differences or odds ratios (OR; with 95% confidence intervals, CI) associated with (1) single, multiple or fractional unit increase of continuous variables (specifically to selected scale locations for curved relationships), (2) index vs. base levels of categorical variables.

Areas under receiver operating characteristic (ROC) curves (AUC) were based on fitting an age- and sex-adjusted logistic regression model between the predictor and the outcome, deriving fitted values (log odds), and using all possible fitted value cutoffs to construct the sensitivity vs. 1–specificity function. Predictors in reverse association with the outcome were multiplied by negative 1 to ensure correct positioning of the function. AUC sizes were nonparametrically compared using the DeLong–Clarke-Pearson method.

The statistical package Stata (version 15.1) was used for data handling and analysis. The significance criterion was set at α = 0.05.

## Results

### Baseline characteristics, comorbidities and cardiovascular medications did not differ significantly between SARS-CoV-2 positive and negative patients

The baseline characteristics and the prevalence of comorbidities or relevant cardiovascular medications of the non-COVID-19 and COVID-19 patients did not differ significantly, as summarized in Table 1.

**Table 1:** Baseline characteristics, cardiovascular risk profile, comorbidities and relevant medications of the non-COVID-19 and COVID-19 groups of the study cohort

Characteristics	Non-COVID-19 (n = 35)	COVID-19 (n = 32)	p value
Baseline characteristics			
Age years (SD)	61.7 (22.27)	59.9 (14.9)	0.71
Female n (%)	18 (51)	16 (50)	1.0
BMI kg/m^2^ (IQR)	23.9 (4.49)	26.7 (6.5)	0.07
Previous medical history			
Hypertension n (%)	18 (51)	12 (38)	0.3
Diabetes mellitus n (%)	6 (17)	7 (21.9)	0.76
Chronic kidney disease n (%)	4 (11)	4 (12.5)	1.0
Coronary artery disease n (%)	1 (3)	2 (6.3)	0.6
Heart failure n (%)	3 (9)	2 (6.3)	1.0
Cancer n (%)	13 (37)	10 (31.3)	0.78
Smoker n (%)	19 (54)	14 (43.8)	0.47
Pulmonary pathology n (%)	5 (14)	9 (28)	0.23
Relevant cardiovascular medications			
ACEI n (%)	6 (17)	2 (6.3)	0.26
ARB n (%)	7 (20)	8 (25)	0.77
CCB n (%)	6 (17)	7 (21.9)	0.76

Continuous variables were described using mean and standard deviation (SD) for symmetric, and median and interquartile range (IQR) for asymmetric distributions. p values are from two-sample t tests (SD), Wilcoxon’s rank-sum test (IQR) or Fisher’s exact test (categorical variables). Pulmonary pathology is defined as COPD, prior pulmonary embolism or pulmonary fibrosis in the anamnesis

*ACEI* angiotensin-converting enzyme inhibitor, *ARB* angiotensin receptor blocker, *BMI* body mass index, *CCB* Ca^2+^ channel blocker, *COPD* chronic obstructive pulmonary disease, *COVID-19* coronavirus disease 2019, *IQR* interquartile range, *SD* standard deviation

Patients tested positive for SARS-CoV-2 infection had a higher need for oxygen supply and reported fever more frequently (Table 2). In the Glasgow coma scale on admission or in the occurrence of dyspnoea, there was no significant difference between the groups. The defined endpoints occurred more frequently in the COVID-19 group (Table 2).

**Table 2:** Clinical characteristics and laboratory parameters of patients in the non-COVID-19 and COVID-19 groups

Characteristics	Non-COVID-19 (n = 35)	COVID-19 (n = 32)	p value
Clinical characteristics			
O_2_ supply L/min (IQR)	0 (0)	0 (9)*	**< 0.01**
Fever n (%)	18 (51)	25 (78)	**0.04**
GCS on admission (SD)	15 (0)	14.6 (2.1)	0.31
Dyspnoea n (%)	17 (49)	19 (59)	0.46
Laboratory parameters			
Leukocytes 10^3^/µL (IQR)	8.7 (4.48)	4.8 (4.2)	**< 0.01**
Platelets 10^3^/µL (IQR)	230.5 (126)	183 (69)	**0.03**
Haemoglobin g/dL (IQR)	12.9 (3.8)	12.8 (4.3)	0.63
CRP mg/L (IQR)	19.5 (42.4)	53.4 (120.2)	**0.03**
PCT ng/mL (IQR)	0.06 (0.13)	0.1 (0.22)	**0.04**
IL-6 pg/mL (IQR)	22.7 (42.3)	33 (95)	0.49
GOT U/L (IQR)	24.5 (14)	41.5 (23)	**< 0.01**
GPT U/L (IQR)	21 (16)	26.5 (20)	**0.03**
GGT U/L (IQR)	34 (67)	62 (89)	0.09
LDH U/L (IQR)	216 (70)	332.5 (228)	**< 0.01**
Bilirubin mg/dL (IQR)	0.5 (0.4)	0.4 (0.25)	0.28
Creatinine mg/dL (IQR)	0.87 (0.43)	0.94 (0.52)	0.53
Ca^2+^ mmol/L (SD)	1.22 (0.04)	1.16 (0.07)	**< 0.01**
Na^+^ mmol/L (SD)	138.5 (3.57)	137.9 (4.5)	0.59
K^+^ mmol/L (SD)	4.4 (0.56)	4.2 (0.67)	0.18
CK U/L (IQR)	70 (58)	60 (95)	0.99
Troponin T ng/L (IQR)	8.2 (26.8)	8.25 (6.7)	0.83
NT-proBNP pg/mL (IQR)	242 (1571)	184.5 (862)	0.57
Myoglobin ng/mL (IQR)	49 (34)	56 (54)	0.39
D-dimer mg/L (IQR)	0.55 (1.13)	1.2 (1.5)	0.07
vWF activity % (IQR)	187.5 (155)	288.9 (102)	**< 0.01**
vWF antigen % (IQR)	195.5 (160)	284.5 (168)	**< 0.01**
Fibrinogen mg/dL (IQR)	368.5 (118.5)	387 (395)	0.73
CNP and furin measurements			
NT-proCNP pmol/L (IQR)	24.8 (18.54)	14.9 (15.97)	**< 0.01**
Furin pg/mL (IQR)	10 (5.9)	10 (91.6)	0.09
Endpoints			
Primary composite endpoint	1 (2.9)	13 (40.6)	**< 0.01**
Need for mechanical ventilation and/or ECMO n (%)	1 (2.9)	12 (38)	**< 0.01**
Death n (%)	1 (2.9)	7 (21.9)	**0.02**

Bold font indicates statistical significance

Continuous variables were described using mean and standard deviation (SD) for symmetric, and median and interquartile range (IQR) for asymmetric distributions. p values are from two-sample t tests (SD), Wilcoxon’s rank-sum test (IQR) or Fisher’s exact test (categorical variables)

*CK* creatine kinase, *CRP* C-reactive protein, *COVID-19* coronavirus disease 2019, *ECMO* extracorporeal membrane oxygenation, *GGT* gamma-glutamyltransferase, *GOT* glutamic oxaloacetic transaminase, *GPT* glutamate-pyruvate transaminase, *IL-6* interleukin 6, *IQR* interquartile range, *LDH* lactate dehydrogenase, *NT-proBNP* amino terminal pro-brain natriuretic peptide, *NT-proCNP* amino terminal pro-C-type natriuretic peptide, *PCT* procalcitonin, *SD* standard deviation, *vWF* von Willebrand factor

*15 of 32 subjects received oxygen, ranging from 1 to 15 L/min, median 12 L/min

### The laboratory tests confirmed pronounced inflammatory state and hypercoagulability in the COVID-19 group

Upon study inclusion, mildly reduced, but still physiologic leukocyte and thrombocyte count in the COVID-19 group could be observed, meanwhile, the haemoglobin values did not differ significantly between the groups (Table 2). The clinical chemistry data showed a significant elevation of inflammatory biomarkers including C-reactive protein (CRP) and procalcitonin (PCT), but not interleukin 6 (IL-6) in the COVID-19 group compared to the non-COVID-19 group (Table 2). There was a slight but significant elevation of glutamic oxaloacetic transaminase (GOT) and glutamic pyruvic transaminase (GPT) in the COVID-19 group compared to non-COVID-19 patients (p < 0.01 and 0.03, respectively) (Table 2). Troponin T and NT-proBNP levels were not significantly different between the two groups. Blood coagulation parameters including the von Willebrand factor (vWF) activity and antigen showed a significant elevation in the COVID-19 group (p < 0.01 for both parameters) (Table 2).

### Discordant changes of NT-proCNP in comparison to other inflammatory biomarkers

**Fig. 2:**
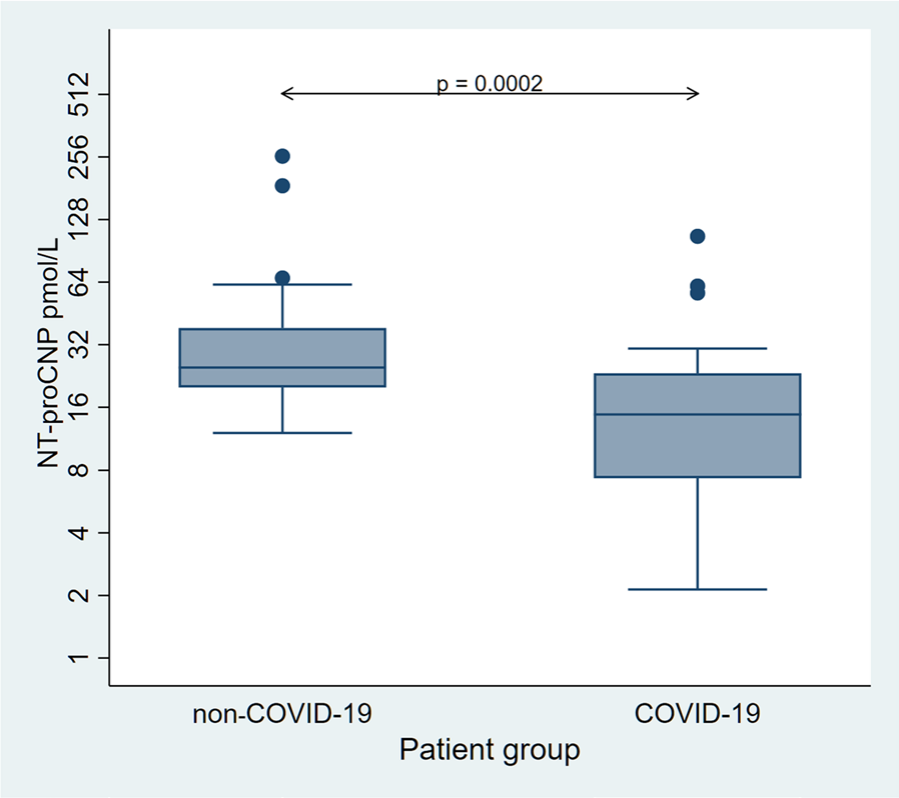
NT-proCNP levels. NT-proCNP levels on admission were significantly decreased in the COVID-19 group (n = 32) compared to the non-COVID-19 group (n = 35). Pairwise between-groups difference reached significance (Wilcoxon’s rank-sum test). *COVID-19* coronavirus disease 2019, *NT-proCNP* amino terminal pro-C-type

NT-proCNP was significantly lower in the COVID-19 group compared to the non-COVID-19 group (p < 0.01 Table 2, Fig. 2). However, there was no significant difference in plasma furin levels between the two groups (Table 2).

### Association of clinical and laboratory parameters with the primary endpoint in the COVID-19 patients

In our further analysis, we exclusively focused on COVID-19 patients, in order to describe the examined parameters and their associations to the defined primary endpoint.

**Fig. 3:**
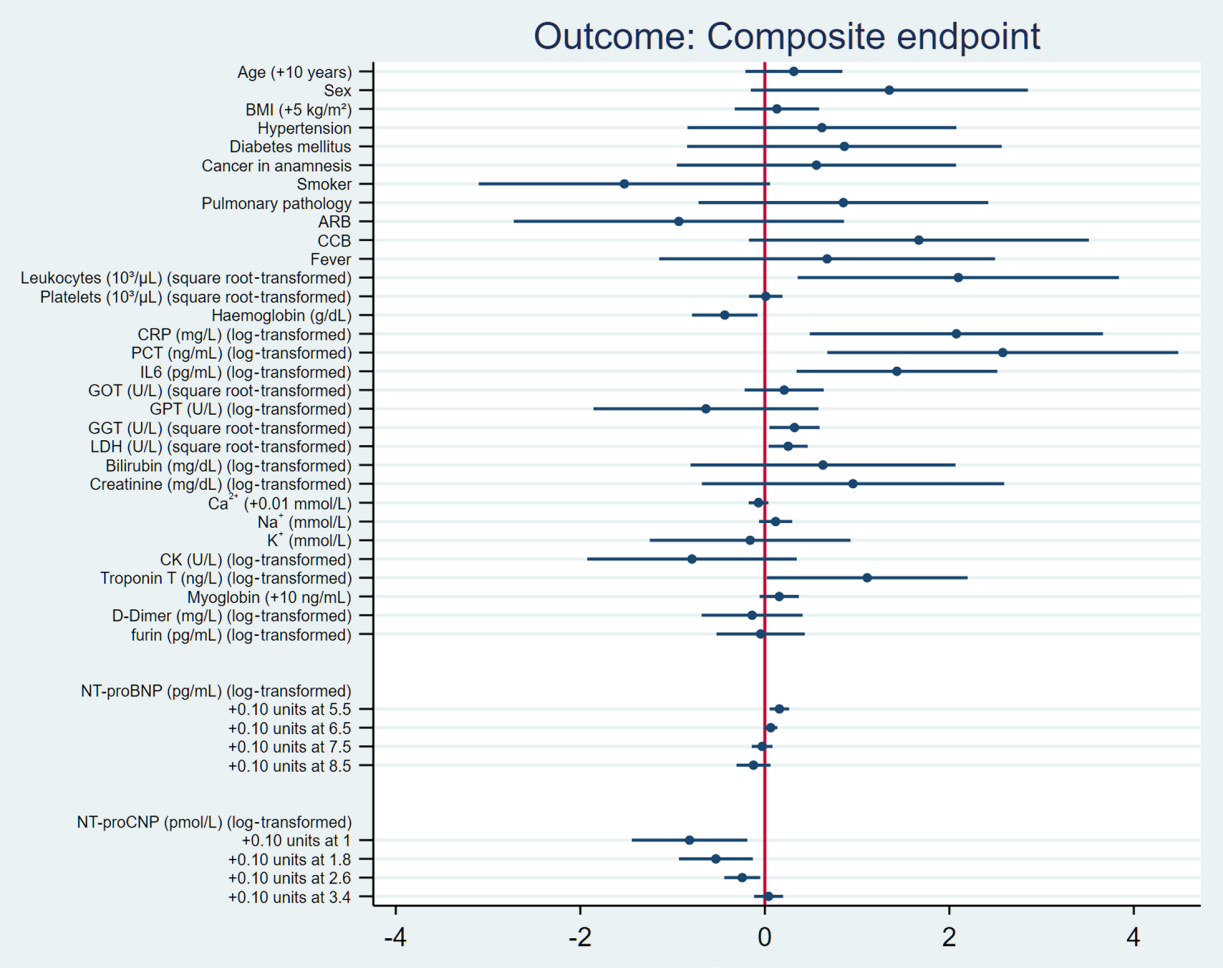
Associations between clinical and laboratory parameters and the composite endpoint (death and/or need for mechanical ventilatory support and/or ECMO) in COVID-19 patients. Scale: log odds difference of the composite endpoint associated with presence vs. absence of categorical predictors, or single-unit (unless indicated otherwise) increase of continuous predictors estimated by simple logistic regression. Markers indicate log odds difference with 95% CI. Some variables were homogenous in the sample (chronic kidney disease, coronary artery disease, heart failure, ACEI, GCS on admission, dyspnoea), confounding by indication (O_2_ supply) or missing in too many subjects (vWF activity, vWF antigen, fibrinogen), thus are not included in this analysis. *ACEI* angiotensin-converting enzyme inhibitor, *ARB* angiotensin receptor blocker, *BMI* body mass index, *CK* creatine kinase, *CRP* C-reactive protein, *COVID-19* coronavirus disease 2019, *ECMO* extracorporeal membrane oxygenation, *GGT* gamma-glutamyltransferase, *GOT* glutamic oxaloacetic transaminase, *GPT* glutamic pyruvic transaminase, *IL-6* interleukin 6, *LDH* lactate dehydrogenase, *NT-proBNP* amino terminal pro-brain natriuretic peptide, *NT-proCNP* amino terminal pro-C-type natriuretic peptide, *PCT* procalcitonin

Higher leukocyte count, CRP, PCT, IL-6, gamma-glutamyl transferase (GGT) and Lactate dehydrogenase (LDH) levels as well as a lower haemoglobin level were significantly and linearly associated with the composite primary endpoint (haemoglobin OR: 0.65, 95% CI: 0.45–0.92, p = 0.016; leukocyte count OR: 8.15, 95% CI: 1.43–46.46, p = 0.018; CRP OR: 7.98, 95% CI: 1.63–39.08, p = 0.01; PCT OR: 13.19, 95% CI: 1.97–88.38, p < 0.01; IL-6 OR: 4.19, 95% CI: 1.41–12.44, p < 0.01; GGT OR: 1.38, 95% CI: 1.05–1.8, p = 0.02; LDH OR: 1.29, 95% CI: 1.04–1.59, p = 0.02) (Fig. 3).

We detected a significant, non-linear association between log-transformed NT-proCNP levels and the primary endpoint. Lower log-transformed NT-proCNP levels on admission were associated with the combined primary endpoint. Adjusted for age and sex, a 0.1-unit increase from a low reference point of log-transformed NT-proCNP such as 1.8 was associated with an OR of 0.482 (95% CI: 0.261–0.890, p = 0.0197); the effect gradually reduced when moving towards higher reference points [e.g., at 2.6, the OR estimate was 0.704 (95% CI: 0.516–0.958, p = 0.0258)], and faded out in the range where NT-proCNP was already high [0.1-unit increase from 3.4: OR = 1.028 (95% CI: 0.871–1.212, p = 0.7465] (see Fig. 3 for unadjusted effect estimates).

In contrast, while investigating the log-transformed NT-proBNP level, a non-significant, non-linear association was seen, showing more pronounced association with the primary endpoint upon elevated log-transformed NT-proBNP levels. Adjusted for age and sex, a 0.1-unit increase from a low reference point of log-transformed NT-proBNP such as 4.5 was associated with an OR of 2.803 (95% CI: 0.962–8.163, p = 0.059); the effect was gradually reduced when moving towards higher reference points [e.g., at 6.5, the OR estimate was 1.667 (95% CI: 0.953–2.917, p = 0.07)], and was further reduced in the range where NT-proBNP was already high [0.1-unit increase from 8.5: OR = 0.992 (95% CI: 0.773–1.272, p = 0.95] (Fig. 3).

### Association of the examined parameters with the secondary endpoints in COVID-19 patients

Decreased haemoglobin levels were significantly and linearly associated with the need for mechanical ventilatory support or ECMO (OR: 0.71, 95% CI: 0.51–0.099, p = 0.04). Leukocyte count, CRP, PCT, IL-6, GGT and troponin T levels as well as CCB in the pharmacotherapy were significantly and linearly associated with the need for mechanical ventilatory support or ECMO: more frequent occurrence or higher levels were associated with the need for mechanical ventilatory support (CCB-therapy OR: 6.43, 95% CI: 1.003–41.2, p = 0.05; leukocyte count OR: 5.59, 95% CI: 1.17–26.69, p = 0.03; CRP OR: 6.42, 95% CI: 1.5–26.72, p = 0.011; PCT OR: 4.52, 95% CI: 1.53–13.33, p < 0.01; IL-6 OR: 2.67, 95% CI: 1.25–5.72, p = 0.01; GGT OR: 1.28, 95% CI: 1.005–1.62, p = 0.045; troponin T OR: 3.03, 95% CI: 1.02–9.01, p = 0.05) (see Additional file 1: Figure S1 for unadjusted effect estimates).

We detected a significant, non-linear association between the decreased serum levels of NT-proCNP or the elevated levels of NT-proBNP and the need for mechanical ventilatory support or ECMO. Adjusted for age and sex, a 0.1-unit increase from a low reference point of log-transformed NT-proCNP such as 1.8 was associated with an OR of 0.434 (95% CI: 0.220–0.853, p = 0.0155); the effect gradually decreased when moving towards higher reference points [e.g., at 2.6, the OR estimate was 0.681 (CI: 0.490–0.0.946, p = 0.0219)], and faded out in the range where the NT-proCNP was already high [0.1-unit increase from 3.4: OR = 1.070 (CI: 0.907–1.261, p = 0.424] (see Additional file 1: Figure S1 for unadjusted effect estimates). Adjusted for age and sex, a 0.1-unit increase from a low reference point of log-transformed NT-proBNP such as 4.5 was associated with an OR of 1.575 (95% CI: 1.128–2.199, p = 0.008); the effect was gradually reduced when moving towards higher reference points [e.g., at 6.5, the OR estimate was 1.250 (CI: 1.032–1.514, p = 0.022)], and was further reduced in the range where the NT-proBNP was already high [0.1-unit increase from 8.5: OR = 0.992 (CI: 0.795–1.239, p = 0.95] (see Additional file 1: Figure S1 for unadjusted effect estimates).

We have seen a significant association between elevated CRP, PCT, IL-6, Troponin T, GGT and LDH levels and death in COVID-19 patients (CRP OR: 3.88, 95% CI: 1.08–13.97, p = 0.04; PCT OR: 3.47, 95% CI: 1.4–8.5, p < 0.01; IL-6 OR: 2.4, 95% CI: 1.18–4.99, p = 0.02; GGT OR:1.47, 95% CI: 1.06–2,04, p = 0.02; LDH OR: 1.3, 95% CI: 1.04–1.6, p = 0.02; Troponin T OR: 3.9, 95% CI: 1.19–12.81, p = 0.03). Further investigation of clinical and laboratory parameters revealed a significant association between decreased haemoglobin and CK level on admission and death in COVID-19 patients (haemoglobin OR: 0.57, 95% CI: 0.36–0.91, p = 0.02; CK OR: 0.05, 95% CI: 0.003–0.84, p = 0.04). On the other hand, the levels of natriuretic peptides were not significantly associated with death (NT-proCNP OR: 0.446, 95% CI: 0.16–1.25, p = 0.12; NT-proBNP OR: 2.36, 95% CI: 0.9–6.16, p = 0.08) (Additional file 1: Figure S1).

Adjusted for age and sex, a 0.01-unit increase from a low reference point of serum Ca^2+^ such as 1.05 was associated with an OR of 0.64 (95% CI: 0.43–0.96, p = 0.03); the effect gradually decreased when moving towards higher reference points [e.g., at 1.1, the OR estimate was 0.77 (95% CI: 0.6–0.98, p = 0.035)], and faded out in the range where the Ca^2+^ was already high [0.1-unit increase from 1.2: OR = 1.1 (95% CI: 0.91–1.34, p = 0.32)] (see Additional file 1: Figure S1 for unadjusted effect estimates).

### Comparison of COVID-19 patients with mild or severe disease course

Two groups of COVID-19 patients were formed based on their need for ICU treatment: mild and severe, as detailed in the Methods section. Baseline characteristics, previous medical history and cardiovascular pharmacotherapy were well-balanced between the two groups (Tables 3 and 4).

**Table 3:** Baseline characteristics, cardiovascular risk profile, comorbidities and relevant medications of the mild and severe COVID-19 patients

Characteristics	Mild COVID-19 (n = 20)	Severe COVID-19 (n = 12)	p value
Baseline characteristics			
Age years (SD)	58.4 (15.7)	62.4 (13.9)	0.47
Female n (%)	12 (60)	4 (33)	0.27
BMI kg/m^2^ (IQR)	26.2 (7)	27.4 (10.6)	0.46
Previous medical history			
Hypertension n (%)	6 (30)	6 (50)	0.29
Diabetes mellitus n (%)	3 (15)	4 (33.3)	0.38
Chronic kidney disease n (%)	4 (11.4)	4 (12.5)	1.0
Coronary artery disease n (%)	2 (10)	0 (0)	0.52
Heart failure n (%)	1 (5)	1 (8.3)	1.0
Cancer n (%)	6 (30)	4 (33.3)	1.0
Smoker n (%)	11 (55)	3 (25)	0.15
Pulmonary pathology n (%)	4 (20)	5 (41)	0.24
Relevant cardiovascular medications			
ACEI n (%)	0 (0)	2 (17)	0.13
ARB n (%)	6 (30)	2 (17)	0.68
CCB n (%)	2 (10)	5 (41.7)	0.07

Continuous variables were described using mean and standard deviation (SD) for symmetric, and median and interquartile range (IQR) for asymmetric distributions. p values are from Wilcoxon’s rank-sum test (continuous variables) or Fisher’s exact test (categorical variables). Pulmonary pathology is defined as COPD, prior pulmonary embolism or pulmonary fibrosis in the anamnesis

*ACEI* angiotensin-converting enzyme inhibitor, *ARB* angiotensin receptor blocker, *BMI* body mass index, *CCB* Ca^2+^ channel blocker, *COPD* chronic obstructive pulmonary disease, *COVID-19* coronavirus disease 2019, *IQR* interquartile range

**Table 4:** Laboratory parameters and clinical characteristics of the mild and severe COVID-19 patients

Characteristics	Mild COVID-19 (n = 20)	Severe COVID-19 (n = 12)	p value
Clinical characteristics			
Dyspnoea n (%)	8 (40)	11 (92)	**< 0.01**
O_2_ supply L/min (IQR)	0 (0)	13.5 (9)	**< 0.01**
GCS on admission (SD)	15 (0)	14 (3.46)	0.19
Fever n (%)	15 (75)	10 (83)	0.68
Laboratory parameters			
Leukocytes 10^3^/µL (IQR)	4.1 (2.6)	7.2 (5.8)	**0.02**
Platelets 10^3^/µL (IQR)	180 (61)	195 (132)	0.24
Haemoglobin g/dL (IQR)	12.9 (2.3)	11.1 (2.5)	**0.03**
CRP mg/L (IQR)	15.5 (56)	160 (162)	**< 0.01**
PCT ng/mL (IQR)	0.07 (0.06)	0.38 (1.1)	**< 0.01**
IL-6 pg/mL (IQR)	13.8 (37.3)	104 (829.6)	**< 0.01**
GOT U/L (IQR)	37.5 (39.5)	44 (15)	0.47
GPT U/L (IQR)	31 (18)	26 (21)	0.45
GGT U/L (IQR)	44 (62)	95 (95)	**0.03**
LDH U/L (IQR)	260 (173)	454 (158)	**< 0.01**
Bilirubin mg/dL (IQR)	0.4 (0.3)	0.4 (0.3)	0.56
Creatinine mg/dL (IQR)	0.92 (0.45)	1.01 (0.73)	0.31
Ca^2+^ mmol/L (SD)	1.17 (0.06)	1.15 (0.09)	0.54
Na^+^ mmol/L (SD)	137 (3.8)	139 (5.3)	0.14
K^+^ mmol/L (SD)	4.16 (0.73)	4.26 (0.56)	0.68
CK U/L (IQR)	72 (123)	60.5 (72.5)	0.25
Troponin T ng/L (IQR)	6.4 (5)	12.9 (23.8)	**< 0.01**
NT-proBNP pg/mL (IQR)	106 (312)	597 (1310)	**< 0.01**
Myoglobin ng/mL (IQR)	64 (33)	89 (50)	0.14
D-dimer mg/L (IQR)	1.17 (1.4)	1.1 (2.1)	0.67
vWF activity % (IQR)	240 (99)	393 (82)	0.06
vWF antigen % (IQR)	277 (143)	414 (3)	**0.01**
Fibrinogen mg/dL (IQR)	303 (119)	688 (234)	**< 0.01**
CNP and furin measurements			
NT-proCNP pmol/L (IQR)	18.8 (11.4)	6.1 (6.6)	**< 0.01**
Furin pg/mL (IQR)	10 (54)	26 (134)	0.69

Bold font indicates statistical significance

Continuous variables were described using mean and standard deviation (SD) for symmetric, and median and interquartile range (IQR) for asymmetric distributions. p values are from two-sample t test (SD), Wilcoxon’s rank-sum test (IQR) or Fisher’s exact test (categorical variables)

*CK* creatine kinase, *CRP* C-reactive protein, *COVID-19* coronavirus disease 2019, *GGT* gamma-glutamyltransferase, *GOT* glutamic oxaloacetic transaminase, *GPT* glutamic pyruvic transaminase, *IL-6* interleukin 6, *IQR* interquartile range, *LDH* lactate dehydrogenase, *NT-proBNP* amino terminal pro-brain natriuretic peptide, *NT-proCNP* amino terminal pro-C-type natriuretic peptide, *PCT* procalcitonin, *vWF* von Willebrand factor

Dyspnoea and the necessity of O_2_-supplementation on study inclusion was significantly more frequent among the severe COVID-19 positive patients (p < 0.01). Inflammatory parameters with predictive value for mortality in critically ill patients (IL-6, CRP, PCT), as well as NT-proBNP were significantly higher in the severe subgroup (p < 0.01 for IL-6, CRP, PCT and NT-proBNP, respectively). The white blood cell count was significantly elevated, and the haemoglobin was significantly decreased in the severe subgroup, but still fell within the physiological range. GGT, lactate dehydrogenase (LDH) and troponin T levels were significantly elevated in the severe subgroup (p = 0.03 for GGT, p ≤ 0.01 for LDH and troponin T). NT-proCNP levels were found significantly lower among patients with severe COVID-19 (p < 0.01).

### Predictive value of natriuretic peptides and conventional inflammatory markers for death in COVID-19

When investigating the predictive value of NT-proCNP for death (AUC: 0.7), there was no sign of better performance over the other inflammatory markers or NT-proBNP, as seen on Additional file 1: Figure S2 (AUC for NT-proBNP: 0.81, AUC for IL-6: 0.91, AUC for CRP: 0.87).

## Discussion

CRP, PCT, IL-6 and NT-proBNP are well known predictors of poor clinical outcome in critically ill patients [3, 11]. Recently, multiple studies have shown that the inflammatory markers CRP, PCT, leukocytes, and IL-6 are important predictors of mortality in COVID-19, too [21–24]. Along with inflammatory biomarkers, the predictive value of elevated NT-proCNP for poor clinical outcome in critically ill patients have also been previously described [11]. Herein, our study is the first to report discordant changes in the levels of NT-proCNP compared to cardiac and conventional inflammatory markers in a patient population with COVID-19. We identified that a decreased NT-proCNP level alone or with simultaneously elevated NT-proBNP and inflammatory parameters in COVID-19 patients on admission is significantly associated with a severe disease course (defined as the composite endpoint of need of mechanical ventilatory support/ECMO or death), thus serves as a good marker of poor outcome in COVID-19.

The detected NT-proCNP levels in the non-COVID-19 group were in line with previous observations and in the range of healthy controls [25].

To resolve the unexpected low levels of NT-proCNP in our COVID-19 positive patient population, one shall study the gene regulation and post-translational processing of CNP, which is, at present, not fully understood [12]. The syntheses of CNP and other natriuretic peptide family members are different. CNP is not stored in granules, unlike ANP or BNP, thus its serum level is always dependent on the production rate. Inflammatory signals, hypoxia and NT-proBNP induce gene transcription of CNP [12]. These parameters are significantly elevated or more common in severe COVID-19, which was also the case in our study cohort. There may be currently unknown regulators of CNP gene expression, possibly leading to a reduced serum level of the molecule. Endothelial damage occurring in deteriorating COVID-19 patients may lead to severe endothelial dysfunction with reduced excretory capacity, which hypothetically could also result in lower NT-proCNP levels. This would be in line with the findings of Dietmann et al., who described decreased NT-proCNP levels in severe malaria cases in children. Similarly to SARS-CoV-2, malaria is an intracellular pathogen and it is characterized by diffuse endothelial damage and dysfunction [26]. In contrast, the level of endothelin-1, a molecule also secreted by the endothelium was found to be elevated [26], which calls into question the strong connection between endothelial damage and reduced endothelial secretory capacity. Furthermore, endothelial damage is associated with “leaky” cell membranes and a net Ca^2+^ influx. At the same time, elevated intracellular Ca^2+^ levels would rather propagate the expression of CNP, as shown by Mendonca et al. [27]. When investigating the serum Ca^2+^ levels of our patients, we found significantly lower levels in COVID-19 than in non-COVID-19 patients and a decreased Ca^2+^ level associated non-linearly and significantly with death in COVID-19 patients. This is an expected finding, as about 50% of critically ill patients develop hypocalcaemia [28]. Nevertheless, our patients did not develop severe hypocalcaemia; the lowest ionised Ca^2+^ level in our cohort was 1.03 mmol/L. Ultimately, endothelial dysfunction resulting in a decreased CNP production seems to be an unlikely explanation for this phenomenon.

Thus, instead of the above explanation, we rather propose that a defect in one of the post-translational steps of the molecule may have caused a relative reduction of NT-proCNP in the severe COVID-19 group. There is one main post-translational step, the cleavage of NT-proCNP from proCNP, which can exclusively be performed by the specific endoprotease furin. According to Wu et al., the presence of furin is crucial in CNP processing [15]. Furin is also essential for the cellular entry of SARS-CoV-2 [18]. Recently, it was proposed that furin could hypothetically be redistributed in SARS-CoV-2 infection, especially in serious COVID-19, resulting in depleted furin activity [29]. As furin is a Ca^2+^-dependent endoprotease, relative hypocalcaemia observed in our COVID-19 group could also add to the previously proposed depleted furin activity, potentially leading to lower NT-proCNP levels in SARS-CoV-2 positive patients. However, hypocalcaemia in critically ill patients is not necessarily bound to a net Ca^2+^ loss, rather to redistribution resulting in intracellular hypercalcaemia [30, 31]. It was demonstrated in parathyreoid cell cultures that the activity of furin is not altered by the extracellular Ca^2+^ level, as no changes were found in furin-mediated proPTH-PTH conversion upon various Ca^2+^ levels [32]. Moreover, the extracellular Ca^2+^ did not alter the RNA expression of furin [33]. As furin plays a key role in the post-translational processing of numerous proteins (albumin, coagulation factors, CNP or parathyroid hormone) [34], its utilisation by SARS-CoV-2 during cellular entry may hypothetically decrease furin bioavailability for the cleavage of such proteins.

In accordance with the results of Ranta et al., who did not find any correlation between serum furin levels and the outcome of sepsis, we could not identify any difference in furin levels between any of the study groups [35]. Nonetheless, whether our measured furin levels truly represent the “whole body furin pool” or rather a relative, stable serum concentration relying on cellular redistribution, is still to be clarified. The activity of accessible furin may also influence its molecular effects [36]. Either way, it is an intriguing hypothesis that SARS-CoV-2 may channel available furin to itself in order to propagate cellular entry of the virus, which then leads to relative furin shortness and the consequent disturbance of furin-mediated post-translational processing of other proteins. If this turns out to be the case, it is of utmost importance to identify what other substrates of furin are affected and how this may influence the course of COVID-19. The novel finding of Wang et al., who demonstrated a SARS-CoV-2 infection-induced alpha-soluble NSF (N-ethylmaleimide-Sensitive Factor) attachment protein (α-SNAP)-mediated furin inhibition in HEK293 (Human Embryonic Kidney 293) and Calu-3 (non-small-cell lung cancer cell line) cells, could strengthen our furin deficiency hypothesis in SARS-CoV-2 infection [37]. Recently, Fajnzylber et al. found that viral load is associated with disease severity and mortality in COVID-19 [38]. Accordingly, the association between disease severity and serum NT-proCNP levels can be explained by a viral load-dependent furin redistribution and consequent unavailability for proteolytic cleavage. It is known that furin is a key molecule for the cellular entry of several other pathogens, too (HIV, Ebola virus, measles virus, RSV) [19]. However, in the literature, no data exist on serum NT-proCNP levels in these infections.

Our findings of a decreased serum NT-proCNP level and its association with a worse disease course in COVID-19 may highlight the clinical importance of CNP in SARS-CoV-2 infection. As CNP is a main regulator of vascular homeostasis, leukocyte activation and platelet reactivity, a decrease in NT-proCNP levels may correspond to all main complications of COVID-19 (acute respiratory distress syndrome, uncontrollable inflammation and thrombotic events) [39]. Nonetheless, it is still to be investigated whether the lack of CNP effects plays a pathophysiological role in COVID-19 deterioration. Further investigations are also needed to demonstrate a direct connection between alterations in the delicate balance of furin homeostasis and the course of infectious diseases, like COVID-19.

## Conclusions

To the best of our knowledge, we are the first to demonstrate discordant changes of serum NT-proCNP levels compared to NT-proBNP and conventional inflammatory markers in severe COVID-19. The significant association between disease progression and reduced NT-proCNP levels was also demonstrated for the first time in COVID-19. Further investigations are required to determine if the decrease in NT-proCNP has any pathophysiological role or therapeutic potential in SARS-CoV-2 infection.

### Limitations

Due to the unavailability of all consecutive patient data and serum samples from the aforementioned time period, and the low number of patients involved, our study is not sufficiently powered to identify strong outcome predictions. Secondly, there may be a fine line between severe and mild COVID-19 cases, and the differentiation system applied by us may be identified as arbitrary. Lastly, serum furin levels remained under detection limit (< 20 pg/mL) in 46 cases. In these patients, according to the recommendations of the manufacturer, the level of furin was uniformly adjusted to 0.0 pg/mL. At the same time, numerous laboratory results and mortality rate in our study are in line with previous observations, suggesting that our findings are not based on a patient selection bias. The observed elevation in biomarker levels of myocardial injury (troponin T) [40], cell death (LDH) [41], myocardial stretch (NT-proBNP) [3] and inflammation (IL-6, CRP, PCT) [42–44], as well as markers of coagulopathy (fibrinogen, vWF) [45, 46] or haematological disorders (leukocytes, platelets, haemoglobin) are all well-described parameters in critically ill patients, as well as in COVID-19 [47]. Furthermore, we could also reproduce the AUC values of others regarding the prognostic value of NT-proBNP, CRP and IL-6 as well [8, 48–50].

## Supplementary Information

**Additional file 1: Figure S1. a) **Associations between clinical and laboratory parameters and the need for mechanical ventilatory support and/or ECMO; **b**) Associations between clinical and laboratory parameters and death. Scale: log odds difference of the composite endpoint associated with presence vs. absence of categorical predictors, or single-unit increase of continuous predictors estimated by simple logistic regression. Markers indicate log odds difference with 95% CI. Some variables were homogeneous in the sample, confounding by indication or missing in too many subjects, thus are not included in this analysis. Abbreviations: ACEI: angiotensin-converting enzyme inhibitor; ARB: angiotensin receptor blocker; BMI: body mass index; CK: creatine kinase; CRP: C-reactive protein; COVID-19: coronavirus disease 2019; ECMO: extracorporeal membrane oxygenation; GGT: gamma-glutamyltransferase; GOT: glutamic oxaloacetic transaminase; GPT: glutamic pyruvic transaminase; IL-6: interleukin 6; LDH: lactate dehydrogenase; NT-proBNP: amino terminal pro-brain natriuretic peptide; NT-proCNP: amino terminal pro-C-type natriuretic peptide; PCT: procalcitonin. **Figure S2.** Receiver operating characteristic curve analyses comparing the diagnostic power of NT-proCNP, NT-proBNP, IL-6 and CRP in predicting death.

## Data Availability

The datasets used and/or analyzed during the current study are available from the corresponding author on reasonable request.
