# Supplementary material for: Decreased level of serum NT-proCNP associates with disease severity in COVID-19
Source: Respir Res. 2023 Jun 29;24:174. doi: 10.1186/s12931-023-02469-4 (PMC10311835; doi:10.1186/s12931-023-02469-4)
Supplement: Supplementary file 1 — Additional file 1: Figure S1. a) Associations between clinical and laboratory parameters and the need for mechanical ventilatory support and/or ECMO; b) Associations between clinical and laboratory parameters and death. Scale: log odds difference of the composite endpoint associated with presence vs. absence of categorical predictors, or single-unit increase of continuous predictors estimated by simple logistic regression. Markers indicate log odds difference with 95% CI. Some variables were homogeneous in the sample, confounding by indication or missing in too many subjects, thus are not included in this analysis. Abbreviations: ACEI: angiotensin-converting enzyme inhibitor; ARB: angiotensin receptor blocker; BMI: body mass index; CK: creatine kinase; CRP: C-reactive protein; COVID-19: coronavirus disease 2019; ECMO: extracorporeal membrane oxygenation; GGT: gamma-glutamyltransferase; GOT: glutamic oxaloacetic transaminase; GPT: glutamic pyruvic transaminase; IL-6: interleukin 6; LDH: lactate dehydrogenase; NT-proBNP: amino terminal pro-brain natriuretic peptide; NT-proCNP: amino terminal pro-C-type natriuretic peptide; PCT: procalcitonin. Figure S2. Receiver operating characteristic curve analyses comparing the diagnostic power of NT-proCNP, NT-proBNP, IL-6 and CRP in predicting death. [file 12931_2023_2469_MOESM1_ESM.docx]

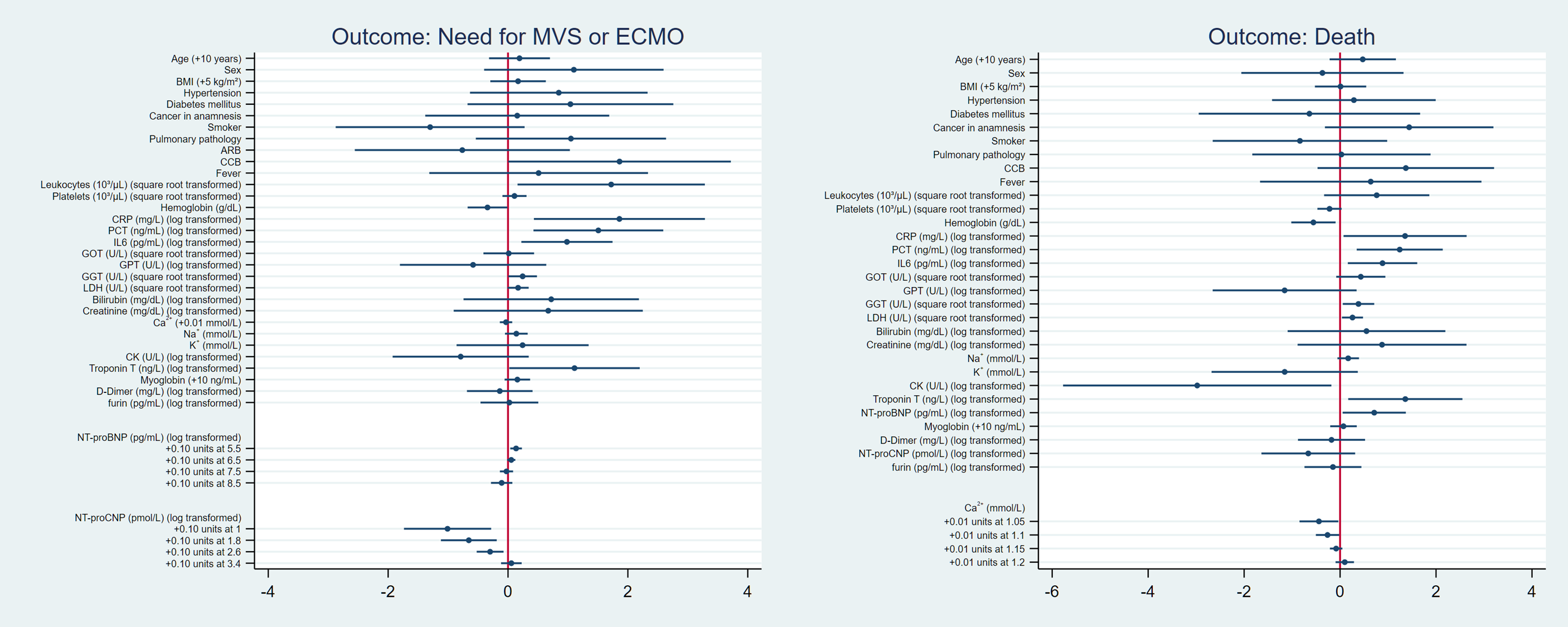


**Additional file 1: figure S1. (a) Associations between clinical and laboratory parameters and the need for mechanical ventilatory support or ECMO; (b) Associations between clinical and laboratory parameters and death.** Scale: log odds difference of the composite endpoint associated with presence vs. absence of categorical predictors, or single-unit (unless indicated otherwise) increase of continuous predictors estimated by simple logistic regression. Markers indicate log odds difference with 95% CI. Some variables were homogenous in the sample (chronic kidney disease, coronary artery disease, heart failure, ACEI, GCS on admission, dyspnoea), confounding by indication (O_2_ supply) or missing in too many subjects (vWF activity, vWF antigen, fibrinogen), thus are not included in this analysis.

Abbreviations: ACEI: angiotensin-converting enzyme inhibitor; ARB: angiotensin receptor blocker; BMI: body mass index; CK: creatine kinase; CRP: C reactive protein; COVID-19: coronavirus disease 2019; ECMO: extracorporeal membrane oxygenation; GGT: gamma-glutamyltransferase; GOT: glutamic oxaloacetic transaminase; GPT: glutamate-pyruvate transaminase; IL-6: interleukin 6; LDH: lactate dehydrogenase; NT-proBNP: amino terminal pro-brain natriuretic peptide; NT-proCNP: amino terminal pro-C-type natriuretic peptide; PCT: procalcitonin.


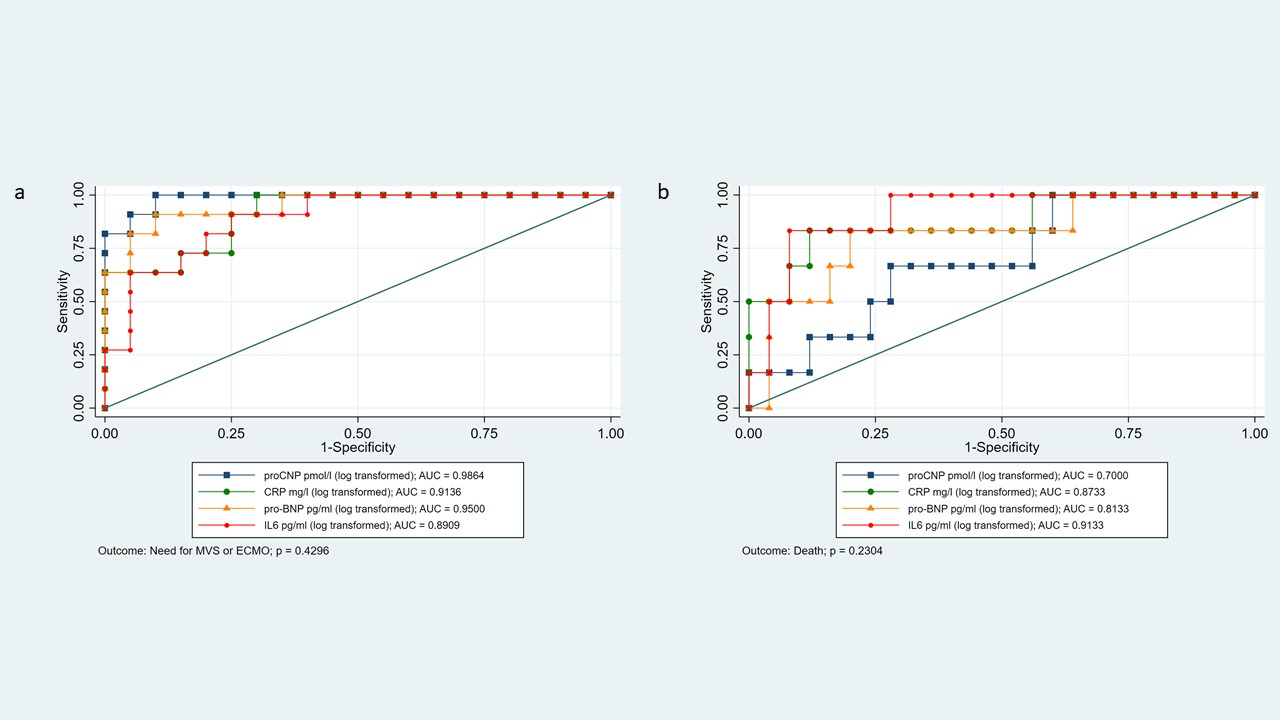


**Additional file 1: figure S2**. Receiver operating characteristic curve analyses comparing the diagnostic power of NT-proCNP, NT-proBNP, IL-6 and CRP in predicting death.
